# Proposing Bromo-Epi-Androsterone (BEA) for Post-Traumatic Stress Disorder (PTSD)

**DOI:** 10.3390/cells14141120

**Published:** 2025-07-21

**Authors:** Coad Thomas Dow, Liam Obaid

**Affiliations:** 1 McPherson Eye Research Institute, University of Wisconsin-Madison, Madison, WI 53705, USA; 2 Keck School of Medicine, University of Southern California, Los Angeles, CA 90089, USA; lobaid@usc.edu

**Keywords:** post-traumatic stress disorder (PTSD), interleukin-1 beta (IL-1β), interleukin 6 (Il-6), tumor necrosis factor alpha (TNFα), high molecular group box 1 protein (HMGB1), blood–brain barrier, damage-associated molecular patterns (DAMP), dehydroepiandrosterone (DHEA), bromo-epi-androsterone (BEA)

## Abstract

Post-traumatic stress disorder (PTSD) has traditionally been viewed as a psychiatric disorder of fear, memory, and emotional regulation. However, growing evidence implicates systemic and neuroinflammation as key contributors. Individuals with PTSD often exhibit elevated blood levels of pro-inflammatory cytokines such as IL-1β, IL-6, TNF-α, and C-reactive protein, indicating immune dysregulation. Dysfunctions in the hypothalamic–pituitary–adrenal (HPA) axis marked by reduced cortisol levels impair the body’s ability to regulate inflammation, allowing persistent immune activation. Circulating cytokines cross a weakened blood–brain barrier and activate microglia, which release additional inflammatory mediators. This neuroinflammatory loop can damage brain circuits critical to emotion processing including the hippocampus, amygdala, and prefrontal cortex, and disrupt neurotransmitter systems like serotonin and glutamate, potentially explaining PTSD symptoms such as hyperarousal and persistent fear memories. Rodent models of PTSD show similar inflammatory profiles, reinforcing the role of neuroinflammation in disease pathology. Bromo-epi-androsterone (BEA), a synthetic analog of dehydroepiandrosterone (DHEA), has shown potent anti-inflammatory effects in clinical trials, significantly reducing IL-1β, IL-6, and TNF-α. By modulating immune activity, BEA represents a promising candidate for mitigating neuroinflammation and its downstream effects in PTSD. These findings support the rationale for initiating clinical trials of BEA as a novel therapeutic intervention for PTSD.

## 1. Introduction

Post-traumatic stress disorder (PTSD) has historically been defined as a psychiatric condition precipitated by trauma, classically characterized by intrusive memories, hyperarousal, and avoidance symptoms. However, emerging research is reshaping this understanding by highlighting a significant biological component to PTSD pathophysiology—namely, chronic neuroinflammation and immune dysregulation [1,2]. Patients with PTSD have been found to exhibit elevated levels of pro-inflammatory cytokines (e.g., interleukin-1β, interleukin-6, tumor necrosis factor-α) and acute-phase reactants like C-reactive protein compared to non-PTSD controls [1]. This heightened inflammatory state is not merely an epiphenomenon; it is implicated in the development and maintenance of core PTSD symptoms and comorbid medical conditions [2,3]. Indeed, chronic low-grade inflammation, particularly within the central nervous system, is increasingly viewed as a key contributor to PTSD’s pathogenesis and its high rates of associated cardiovascular, metabolic, and neurodegenerative disorders [2].

Much of the impetus for reconceptualizing PTSD as a neuroinflammatory disease stems from studies in US veterans. Veterans with combat-related PTSD show robust evidence of immune activation; for example, a Department of Veterans Affairs cohort study (N = 735) found that individuals with current PTSD had significantly higher inflammatory markers than those with remitted or no PTSD [3]. These findings suggest that active PTSD is accompanied by a systemic pro-inflammatory shift, which normalizes when symptoms abate. Parallel research using multi-omics approaches in war-zone-exposed veterans has identified reproducible molecular signatures of PTSD involving upregulated inflammation-related pathways and immune dysregulation [4]. Such studies, often led by collaborative teams at the Department of Veterans Affairs, Department of Defense, NIH, and leading academic centers, underscore a growing consensus that PTSD’s clinical manifestations are intertwined with biological processes of chronic inflammation. Preclinical models further reinforce this paradigm, as exposure to severe stress in animals triggers neuroinflammatory cascades—including microglial activation and cytokine release—that produce anxiety- and fear-related behaviors analogous to PTSD [1].

Viewing PTSD through the lens of neuroinflammation carries significant clinical implications. It provides a mechanistic framework to explain why PTSD sufferers, particularly combat veterans, face elevated risks of immunologically mediated conditions from autoimmune disorders to accelerated cardiovascular disease [2]. It also opens the door to novel interventions: therapies targeting immune pathways (e.g., anti-inflammatory or immunomodulatory agents) and biomarkers of inflammation are being explored as adjuncts to conventional psychotherapeutics [2]. This evolving framework situates PTSD not only as a disorder of the mind, but as a complex neuroimmune condition, a perspective that may fundamentally influence future research and treatment approaches [3,5]. Considering this paradigm shift, this study will examine the evidence linking chronic inflammation to PTSD, elucidate the underlying neuroimmune mechanisms, and discuss the potential for an immune-focused strategy to improve outcomes for those suffering from this often-refractory disorder.

## 2. Mechanisms of Neuroinflammation in PTSD

The pathogenesis of PTSD is increasingly viewed as sustained neuroinflammatory signaling where stress-induced immune dysregulation leads to persistent alterations in central nervous system (CNS) homeostasis. A key mediator in this process is high mobility group box 1 (HMGB1), a nuclear protein that, when released extracellularly during cellular stress or injury, functions as a damage-associated molecular pattern (DAMP). HMGB1 is passively released by necrotic cells or actively secreted by immune cells in response to trauma, where it acts as a potent amplifier of innate immune signaling [6].

Once in the extracellular milieu, HMGB1 interacts with several pattern recognition receptors, notably Toll-like receptor 4 (TLR4) and the receptor for advanced glycation end products (RAGE). Binding of HMGB1 to TLR4 initiates a signaling cascade that activates NF-κB and triggers transcription of the aforementioned pro-inflammatory cytokines such as IL-1β, IL-6, and TNF-α [7,8]. Concurrently, HMGB1 engagement of RAGE, a multi-ligand receptor expressed on neurons, endothelial cells, and microglia promotes cellular adhesion, migration, and sustained inflammatory responses via MAPK and JAK/STAT pathways [9].

The HMGB1–TLR4–RAGE signaling axis has been implicated in microglial priming and activation, a hallmark of chronic neuroinflammation observed in PTSD models. Activated microglia perpetuate the inflammatory state by producing reactive oxygen species (ROS), nitric oxide, and additional cytokines that further exacerbate neuronal dysfunction and synaptic remodeling [10]. Notably, preclinical studies have shown that HMGB1 is upregulated in brain regions associated with fear and emotion regulation, including the amygdala and hippocampus, following traumatic stress [11]. In parallel, increased expression of TLR4 and RAGE has been observed in the prefrontal cortex and hippocampus of rodent models subjected to chronic stress paradigms [12].

Another critical outcome of HMGB1–mediated signaling is the compromise of the blood–brain barrier (BBB). HMGB1 and pro-inflammatory cytokines increase BBB permeability, allowing peripheral immune cells and inflammatory mediators to infiltrate the CNS, thereby establishing a feed-forward loop of neuroimmune activation [13]. This phenomenon is particularly relevant to PTSD, as increased BBB permeability has been associated with both systemic inflammation and the onset of cognitive and affective symptoms.

HMGB1 serves as a pivotal damage-associated molecular pattern (DAMP) linking trauma to innate immune activation in the CNS. Through its interactions with TLR4 and RAGE, HMGB1 orchestrates a cascade of neuroinflammatory events including microglial activation, cytokine secretion, and BBB disruption that underlie the neuropathophysiological changes seen in PTSD.

Moreover, additional molecular pathways implicated in PTSD include cyclooxygenase-2 (COX-2). COX-2 is an inducible enzyme responsible for the synthesis of pro-inflammatory prostaglandins in the central nervous system. Elevated COX-2 expression has been reported in preclinical models of stress and fear learning, where it contributes to both synaptic plasticity and inflammation [14]. Inhibition of COX-2 has been shown to reduce anxiety-like behavior and modulate amygdala reactivity, suggesting that prostaglandin signaling may be a key amplifier of the neuroimmune response in PTSD [15].

In parallel, altered redox metabolism, particularly involving glucose-6-phosphate dehydrogenase (G6PD), has emerged as another contributor to PTSD-related pathology. G6PD is the rate-limiting enzyme in the pentose phosphate pathway and a primary source of NADPH which is essential for both antioxidant defense and immune cell function. Reduced G6PD activity can lead to increased oxidative stress and impaired resolution of inflammation [16]. This is especially relevant in PTSD where both oxidative burden and immune dysfunction are elevated. Diminished NADPH availability may also impair the immune-modulating function of neurosteroids such as DHEA, which relies on redox-sensitive mechanisms to suppress inflammatory pathways, including those involving HMGB1 and TLR4.

Taken together, these findings suggest that PTSD is a psychiatric manifestation of chronic neuroimmune and metabolic dysregulation wherein uncontrolled inflammation, oxidative stress and aberrant prostaglandin signaling converge. This reconceptualization of PTSD opens the door for therapeutic strategies targeting these biological pathways. In subsequent sections, we will explore inflammatory cytokines in PTSD, the anti-inflammatory role of DHEA, and the emerging promise of its synthetic analog, bromoepiandrosterone (BEA), as a candidate for immune modulation in PTSD.

## 3. Mitochondrial Dysfunction in PTSD

Mitochondrial dysfunction is associated with psychiatric disorders [17] and is increasingly recognized as a central pathophysiological feature of PTSD, contributing not only to impaired neuroplasticity and cognitive dysfunction but also to the chronicity of neuroinflammatory responses [18]. Mitochondria are critical regulators of neuronal energy production, redox homeostasis, calcium buffering, and apoptotic signaling. In individuals with PTSD, as well as in stress-exposed animal models, a consistent pattern emerges: impaired mitochondrial oxidative phosphorylation (OXPHOS), elevated production of reactive oxygen species (ROS), and reduced expression of key transcriptional co-activators such as peroxisome proliferator-activated receptor gamma coactivator-1 alpha (PGC-1α), a master regulator of mitochondrial biogenesis [18,19].

This decline in mitochondrial respiratory function not only compromises ATP synthesis but also promotes oxidative stress through excess ROS generation, which damages mitochondrial DNA, lipids, and proteins, exacerbating mitochondrial inefficiency in a feed-forward manner [19]. In PTSD, this vicious cycle appears further reinforced by a shift in mitochondrial dynamics toward fission (mediated by proteins like DRP1) rather than fusion (regulated by proteins such as MFN1/2 and OPA1), leading to fragmented and less functional mitochondria [19]. Fragmentation is often associated with increased susceptibility to cell death and decreased mitochondrial resilience under stress.

**Figure 1:**
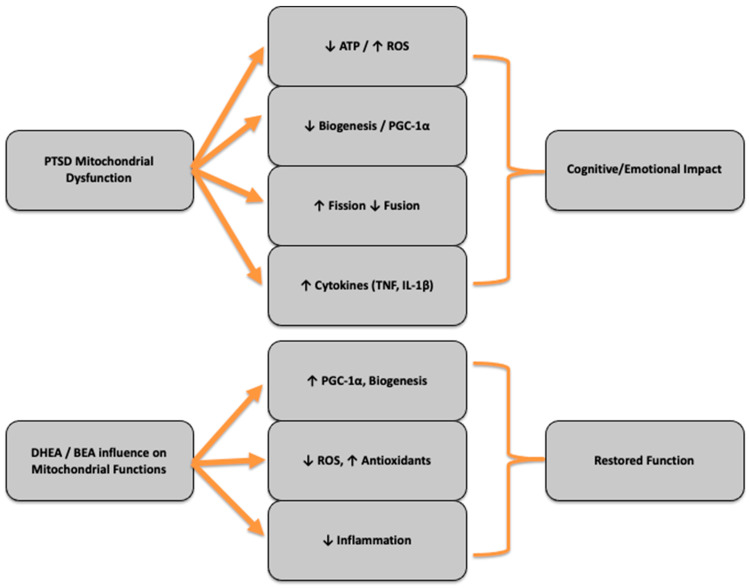
Mitochondrial Dysunction in PTSD. This figure shows the myriad physiologic alterations induced by mitochondrial dysfunction (**top**) as well as the proposed reversal of those alterations and restored function via neuromodulation imparted by DHEA and BEA (**bottom**).

Dysfunctional mitochondria also contribute to dysregulated mitophagy: the selective autophagic removal of damaged mitochondria. In PTSD models, mitophagy appears impaired or insufficient, resulting in the accumulation of dysfunctional mitochondria that continue producing ROS and perpetuating neuroinflammation [19]. These cellular stress signals can activate innate immune pathways, including NF-κB and inflammasome complexes, leading to elevated levels of the pro-inflammatory cytokines IL-1β, TNF-α, and IL-6 [18]. This low-grade, chronic inflammation not only exacerbates neuronal injury but also interferes with neurogenesis and synaptic remodeling, which are critical for recovery from psychological trauma. Moreover, the hippocampus and prefrontal cortex, regions involved in memory, emotional regulation, and executive function appear especially vulnerable to mitochondrial dysfunction. In these areas, impaired mitochondrial activity has been linked to dendritic spine loss, synaptic pruning abnormalities, and a reduced capacity for long-term potentiation (LTP), correlating with the cognitive and emotional symptoms of PTSD [19]. These ramifications of mitochondrial dysfunction in PTSD are seen in the top portion of Figure 1.

## 4. Inflammatory Cytokines in PTSD

A 2015 meta-analysis examined inflammatory markers in PTSD and found elevated levels of IL-1β, IL-6, TNF-α, and interferon-γ among individuals with PTSD compared to controls. Importantly, IL-1β was identified as a potential biomarker of illness duration, while IL-6 was linked to symptom severity, suggesting distinct roles for these cytokines in disease progression and clinical expression [20]. In a follow-up study, the same research group further explored the relationship between inflammatory markers and PTSD phenotypes in a large cohort. They confirmed that elevated IL-6 and C-reactive protein (CRP) levels were associated with PTSD severity and chronicity, particularly among individuals with heightened re-experiencing symptoms and prolonged symptom duration. The follow-up study also provided evidence for the state–trait nature of inflammation in PTSD with IL-6 emerging as a consistent marker across different disease stages. Together, these studies underscore the role of systemic inflammation in PTSD and support the use of IL-1β, IL-6, and CRP as potential biomarkers for disease monitoring and therapeutic targeting [21].

## 5. DHEA and PTSD

Dehydroepiandrosterone (DHEA), along with its sulfated form DHEAS (hereafter, collectively referred to as DHEA), comprises the most prevalent class of steroid hormones found in humans. The secretion of DHEA undergoes distinct developmental changes throughout the human lifespan. During fetal development, elevated levels of DHEA are produced by the adrenal fetal zone [22]. Following birth, these levels decline significantly over the first several months and remain suppressed until adrenarche, which typically occurs between ages six and eight. At that time, production shifts to the zona reticularis of the adrenal cortex, resulting in a gradual increase in circulating levels [23,24]. DHEA concentrations in plasma and cerebrospinal fluid reach their apex in the mid-twenties and then exhibit a progressive decline with advancing age, with levels falling to roughly 20% of their peak by the late 60s—a period coinciding with the rising prevalence of many age-related diseases [25,26,27].

Mounting evidence links dysregulation of DHEA levels to the pathophysiology of various neuropsychiatric disorders. In the central nervous system, DHEA contributes to several critical processes, including neuroprotection, neurite extension, neurogenesis, and neuronal survival. It also influences apoptosis, modulates catecholamine synthesis and release, and exhibits antioxidant, anti-inflammatory, and anti-glucocorticoid properties [28].

There is an increased understanding of DHEA in the context of PTSD; studies demonstrate that individuals with PTSD often exhibit elevated levels of DHEA and a reduced cortisol/DHEA ratio, suggesting a neuroendocrine shift that may reflect an adaptive or compensatory response to chronic stress [29]. Further, higher DHEA levels may be associated with psychological resilience and neuroprotection, potentially modulating the impact of trauma on brain function and emotional regulation [29]. This line of research positions DHEA as both a biomarker and a possible therapeutic target in stress-related psychopathology [28,30].

Supplementation with exogenous DHEA may produce unwanted consequences, including estrogenic or androgenic side effects [31], and although DHEA can impart antioxidant effects [32], at larger doses, it is pro-oxidant [33,34].

## 6. Bromoepiandrosterone (BEA)

16α-Bromoepiandrosterone (BEA) is a synthetic analog of DHEA, structurally modified to eliminate the androgenic and pro-oxidant effects observed with high-dose DHEA. These changes make BEA a promising therapeutic candidate, offering the benefits of DHEA without the associated risks of hormonal overstimulation and oxidative stress [35,36].

BEA, previously known as HE2000, has significantly enhanced biological potency compared to its parent, DHEA. It acts as a strong inhibitor of glucose-6-phosphate dehydrogenase (G6PDH), displaying approximately 60 times greater efficacy than DHEA in enzyme inhibition [37,38]. Originally developed in the late 1990s for its potential in managing infectious diseases, BEA has since been evaluated in nine clinical studies involving a total of 228 individuals, addressing conditions such as HIV, malaria, and hepatitis [37,38,39,40,41]. In recognition of its therapeutic promise, the US FDA granted BEA investigational new drug (IND) status in 1999 for the treatment of HIV/AIDS, where it demonstrated encouraging clinical results [42,43,44]. Beyond antiviral and antibacterial activity, BEA exhibits robust immunomodulatory properties, notably in reducing pathological inflammation [35,45]. Collectively, this has led to its investigation as a candidate therapy for *Mycobacterium tuberculosis*, the leading cause of death from infectious disease globally [46].

Immunologically, BEA mimics the Th1-skewing effects of its parent compound, DHEA, aiding in the restoration of Th1/Th2 balance, a shift that is commonly observed with aging. Rebalancing these immune responses may help mitigate age-related declines in vaccine responsiveness, susceptibility to infections and increased prevalence of autoimmune diseases [47,48,49]. Earlier human trials utilized an intramuscular oil-based formulation of BEA, which was associated with local adverse events such as mild to moderate pain and tissue induration at the injection site [39,43]. A newer, water-soluble formulation has since been developed and is expected to reduce these local reactions substantially [50].

Positive experience with DHEA gives reason to believe that its halogenated analog, BEA, may be a modulator of mitochondrial dysfunction within the context of PTSD through several biological effects [51].

In preclinical models and human studies, DHEA has been observed to counteract mitochondrial deficits by dampening inflammatory cytokines and bolstering antioxidant systems. Notably, in populations with PTSD, mitochondrial-related metabolic shifts across glycolysis, fatty acid oxidation, and the TCA cycle underscore the pronounced mitochondrial stress’ contribution to PTSD. DHEA protects against oxidative mitochondrial damage [52]. Its antioxidant effects likely mitigate the inflammatory cascades, indirectly preserving mitochondrial integrity and function [53]. A critical mechanism is the activation of mitochondrial biogenesis via the PGC-1α → NRF-1/2 → TFAM pathway as seen in the lower portion of Figure 1. PGC-1α is acknowledged as the master regulator of mitochondrial renewal and energy homeostasis [54]. In liver and other tissues, DHEA has been experimentally shown to upregulate PGC-1α, NRF-1, and TFAM—leading to higher ATP production, increased mitochondrial DNA content, and reduced oxidative stress [55]. This cascade mirrors the fundamental biology of mitochondrial quality control, linking biogenesis and clearance in a balanced cycle [56]. Furthermore, DHEA’s influence extends into neurochemical domains: it modulates GABAergic and glutamatergic signaling, neural circuits deeply intertwined with cognition and stress resilience. While DHEA clinical trials show modest cognitive improvement, the neurochemical modulation offers a plausible biological bridge to its stress-buffering effects, providing the reason to believe BEA, the potent, water-soluble, non-androgenic DHEA analog may intervene more successfully in these pathways. Preclinical data indicate that BEA mirrors DHEA’s anti-inflammatory, metabolic, and mitochondrial-enhancing properties—without androgenic side effects. In models noted for chronic inflammation and metabolic dysregulation, BEA reportedly restores cytokine balance and fortifies mitochondrial integrity [41,42], suggesting a unique potential in PTSD-linked mitochondrial derangements.

A logical progression in BEA’s development is to investigate its effects in validated animal models of PTSD. There are multiple existing animal models of PTSD (Table 1). A comprehensive review examined the strengths and limitations of commonly used rodent models, such as single prolonged stress (SPS), stress–re-stress (S-R), and predator-based paradigms in replicating human PTSD pathology [57].

Although these models provide valuable insights into neuroendocrine responses, genetic predispositions, and potential therapeutic targets, they face challenges in capturing the full complexity of PTSD, particularly in terms of ethological relevance and translational validity. While some of these models primarily assess behavioral phenotypes, others incorporate analyses of mechanistic pathways underlying neuroinflammation, including the role of proinflammatory cytokines and innate immune activation. Of the models referenced in Table 1, several tested for, and demonstrated elevated IL-1β, TNF-α, and IL-6 [63,66,70,71].

**Figure 2:**
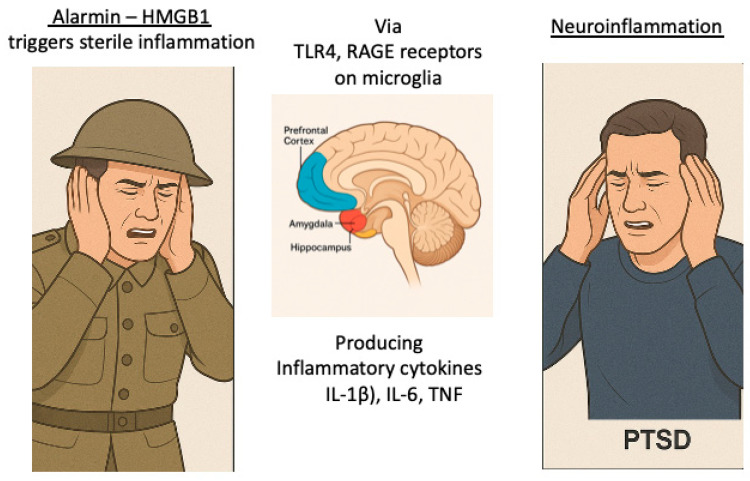
Alarmins generated from psychologic trauma initiate a cascade resulting in neuroinflammation, microglial activation and oxidative stress. Impacting the amygdala, hippocampus and prefrontal cortex, it produces the behavior and memory manifestations of PTSD.

A key discovery regarding co-occurrence of neuroinflammation and microglial activation came from Alzheimer’s disease research; these findings illustrate that Toll-like receptor (TLR) activation is required for resultant tauopathy of Alzheimer’s [72]. Toll-like receptors 2 and 4 (TLR2 and TLR4) are pattern recognition receptors that detect both damage-associated molecular patterns (DAMP- referenced earlier) and pathogen-associated molecular patterns (PAMPs). In the brain, activation of these receptors triggers the NF-κB signaling pathway, leading to increased expression of proinflammatory cytokines such as IL-1β, TNF-α, and IL-6. In models of PTSD, activation of TLR2 and TLR4 has been observed following psychological stress, implicating innate immune signaling as a contributor to chronic neuroinflammation [70]. Figure 2 graphically reflects the inflammatory cascade producing neuroinflammation that results in PTSD. 

## 7. Discussion

DHEA has been shown to function as a negative allosteric modulator of GABA-A receptors [73] and a positive modulator of NMDA receptor function [74]. Given the structural similarity of BEA to DHEA, it is plausible that BEA may exhibit neuromodulatory actions on glutamatergic and GABAergic systems relevant to PTSD pathophysiology [75]. These actions shift the excitatory/inhibitory balance toward greater cortical excitability, which may facilitate cognitive flexibility and emotional processing. This is particularly relevant in PTSD, where dysfunctional inhibitory tone and heightened glutamatergic signaling are core features of the disorder’s neurobiology. Although direct studies on BEA’s receptor interactions remain limited, its structural and functional analogies to DHEA suggest that it may exert similar modulatory effects, potentially contributing to its observed behavioral and immunomodulatory benefits [76].

Current treatment guidelines for PTSD primarily emphasize psychotherapeutic approaches, such as exposure therapy and cognitive restructuring. However, these standard interventions often fall short, with up to 50% of patients not responding in clinical studies [77]. Moreover, many Veterans from Operation Enduring Freedom, Operation Iraqi Freedom, and Operation New Dawn (OEF/OIF/OND) show reluctance to participate in traditional mental health care [78]. The use of complementary and alternative medicine (CAM), particularly for stress- and trauma-related symptoms, has grown rapidly in the United States; research indicates that Veterans with PTSD may be more open to CAM therapies than those without PTSD [79]. These findings collectively support the pursuit of innovative strategies that address the underlying mechanisms of PTSD.

A wide range of pharmacological treatments have been evaluated in PTSD, from repurposed antidepressants and blood pressure medications to novel neurohormonal agents and psychedelics. As PTSD is associated with oxidative stress, antioxidant therapy focused on mitochondrial function has shown limited benefit [80]. Traditional drugs like SSRIs and SNRIs offer modest relief for many (especially civilian PTSD), addressing serotonin/norepinephrine dysregulation [81]. Adrenergic agents (prazosin, propranolol) target the overactive sympathetic nervous system, with prazosin helping some patients’ sleep [82] and propranolol showing potential in memory modification [83]. Glutamatergic drugs are emerging as powerful (e.g., ketamine for rapid symptom control) [84], D-cycloserine to enhance therapy [85]), highlighting the role of learning and extinction pathways in recovery. The breakthrough with MDMA-assisted therapy demonstrates that profound symptom remission is achievable by pharmacologically facilitating emotional processing—an integration of drug and therapy action on PTSD’s core pathology [86]. Meanwhile, drugs like cannabinoids and neurosteroids explore alleviating specific PTSD symptoms (nightmares, hyperarousal) by mimicking or enhancing the brain’s natural modulators (endocannabinoids, GABAergic neurosteroids) [87]. Finally, and the space into which BEA may fit, are investigational approaches addressing inflammation and oxidative stress; interventions that recognize PTSD’s systemic impact beyond neurotransmitters [88]. One such drug is trilostane, an inhibitor of 3β-hydroxysteroid dehydrogenase, causes an increase in neurosteroids; a veterinary product, it may have utility in neuroinflammation [89].

PTSD often coexists with both depression and seizures. Approximately 10–20% of individuals with PTSD develop comorbid seizure disorders, especially non-epileptic or stress-induced seizures [90]. For example, in depression, neurosteroid levels and restoration of these levels is associated with anxiolytic and antidepressant effects. Approximately 10–20% of individuals with PTSD develop comorbid seizure disorders, especially non-epileptic or stress-induced seizures [90]. This underlies the FDA approval of brexanolone (a metabolite of DHEA) for postpartum depression, and ongoing investigation of its effects in major depressive disorder (MDD) [91,92].

PTSD individuals with coexisting MDD and epilepsy or seizure-like events further complicate treatment and reduce quality of life. Several pharmacologic agents evaluated for PTSD also exhibit therapeutic effects on these comorbidities. Selective serotonin reuptake inhibitors (SSRIs) and serotonin-norepinephrine reuptake inhibitors (SNRIs)—notably sertraline, paroxetine, and venlafaxine—are first-line treatments for both PTSD and MDD. In clinical trials, these agents improved not only PTSD symptom clusters but also depressive symptoms when present [90]. Ketamine, an NMDA receptor antagonist, has demonstrated rapid antidepressant effects alongside PTSD symptom relief in treatment-resistant populations [93]. MDMA-assisted therapy, recently validated in two Phase 3 trials, also significantly reduced depressive symptoms in PTSD patients, suggesting that its serotonin- and oxytocin-mediated mechanisms may address both conditions simultaneously [86].

For patients with co-occurring epilepsy, the anticonvulsant topiramate has shown dual benefit: in addition to controlling seizures, it reduced PTSD re-experiencing symptoms and nightmares in small clinical trials [84,93]. Lamotrigine, another antiepileptic with antidepressant properties, improved anger and intrusive symptoms in PTSD while also stabilizing mood, particularly in patients with bipolar depression [94]. While propranolol and prazosin target PTSD’s adrenergic dysregulation, they are not established treatments for depression or epilepsy, though prazosin may reduce suicidal ideation in veterans with PTSD [95]. Emerging anti-inflammatory and neuroprotective agents like N-acetylcysteine (NAC) have shown promise in PTSD with comorbid substance use and depression, possibly through modulation of oxidative stress and glutamatergic balance [96]. Overall, these overlapping therapeutic effects support an integrative approach to PTSD treatment that considers and targets comorbid depressive and epileptiform conditions when present.

BEA may find a place in treatment of these diseases with neuroinflammation as reflected in its suggested use in perioperative neurocognitive disorders (PND) [97] and in stiff person syndrome (SPS) [98].

The next step for BAE would be an animal model trial that would support a randomized, double-blinded, placebo-controlled clinical trial of individuals meeting DSM-5 criteria for PTSD is contemplated. The projected study would assess whether adding BEA offers additional benefits over conventional PTSD treatment alone. Outcomes measured would be improvement in validated PTSD psychological tools and decrease in the pro-inflammatory cytokines.

This proposed trial is grounded in the evolving understanding of PTSD as a neuroinflammatory disorder and the unique neuroendocrine capacity of BEA to enhance immunity and dampen non-productive inflammation. Ultimately, the goal is to improve outcomes, not just for combat veterans, but all those living with PTSD by addressing not only the psychological scars of trauma but also the invisible inflammatory wounds that accompany chronic stress.

## Figures and Tables

**Table 1:** PTSD animal model summary [58,59,60,61,62,63,64,65,66,67,68,69,70,71].

PTSD Animal Model Summary			
Model	Description	PTSD Feature Modeled	References
Fear Conditioning	Tone-shock pairing	Persistent fear, amygdala circuits	Flandreau [58] Maren [59]
Stress-Enhanced Fear Learning (SEFL)	Stressor precedes conditioning	Fear over- consolidation	Rau [60] Pennington [61]
Single Prolonged Stress (SPS)	Restraint → swim → ether	HPA dysregulation, extinction deficits	Liberzon [62] Knox [63]
Social Isolation/ Chronic Unpredictable Stress	Isolation and/or unpredictable mild stress	Impaired extinction, reduced neurosteroids	Locci [64] Serra [65]
Arousal-Based Individual Screening (AIS)	Trauma + screening for hyperarousal	Individual vulnerability/resilience	Torrisi [66] Ritov [67]
Sleep/Physiologic Models	Disrupted REM/NREM after stress	Sleep fragmentation, hyperarousal	Grafe [68] Jung [69]
TLR2/PGE2 in Social Defeat Stress	10-day intruder- aggressor exposure	Social avoidance, anxiety-like behavior	Nie [70] Ishikawa [71]

## Data Availability

No new data were created or analyzed in this study.
